# Communicating Bronchopulmonary Foregut Malformation Type IB: Diagnostic and Surgical Challenges

**DOI:** 10.1055/s-0041-1740321

**Published:** 2021-12-13

**Authors:** Bhushanrao Jadhav, Ranjithatharsini Vaseeharan, Prabhu Sekaran, Semiu Eniola Folaranmi, Karim Awad

**Affiliations:** 1 Department of Paediatric Surgery, Noah's Ark Children's Hospital for Wales, Cardiff, United Kingdom of Great Britain and Northern Ireland; 2 Department of Paediatric Surgery, Ain Shams University, Cairo, Cairo, Egypt

**Keywords:** esophageal lung, esophageal atresia and tracheoesophageal fistula, communicating bronchopulmonary foregut malformations

## Abstract

Communicating bronchopulmonary foregut malformations (CBPFM) are extremely rare. We present a complex case of type IB CBPFM with esophageal atresia and distal tracheoesophageal fistula (EA/TOF), duodenal atresia/annular pancreas (DA/AP), and intestinal malrotation who underwent primary repair for EA/TOF on day 3. Bilious aspirates on day 8 prompted an upper gastrointestinal (GI) contrast revealing a duodenal obstruction and communication between the right lung lower lobe and the esophagus (T8-T9 level). DA/AP and malrotation were repaired by a gastrojejunostomy and Ladd's procedure. A repeat contrast swallow identified a 2nd communication from the esophagus into the right lower lobe (T5-T6 level) raising the suspicion of a recurrent TOF. Computed tomography (CT) thorax confirmed above findings with an anomalous blood supply to right lung. An exploratory thoracotomy identified a three-lobed lung. However, the lower lobe was enlarged and connected in two separate locations to the esophagus. The child recovered after the disconnection of the esophageal connections and partial right lower lobectomy. CBPFM are extremely rare anomalies requiring a high index of suspicion, use of an upper GI contrast series, and CT scans for diagnosis. The treatment of choice is resection of the affected lung and disconnection of the esophageal communications.

## Introduction

Communicating bronchopulmonary foregut malformations (CBPFM) are extremely rare communicating lesions between the respiratory tract and foregut. 1 The early diagnosis is difficult. Delayed recognition can lead to considerable complications. 2 Srikanth et al divided CBPFM into four major groups after a review of 57 cases. 3 CBPFM group I consists of anomalies associated with esophageal atresia and tracheoesophageal fistula with two subdivisions—IA, total sequestered lung in communication with the foregut, and IB, a sequestered segment or lobe of lung in communication with the foregut. Group II has absent main stem bronchus and the total sequestered lung usually the right, communicating with the lower esophagus. Group III has an isolated part of the lung in communication with the esophagus. Group IV has a communication between the normal bronchial system and the esophagus. 3 We present a complex case of CBPFM falling in type IB subgroup with esophageal atresia and distal tracheoesophageal fistula (EA/TOF), duodenal atresia/annular pancreas (DA/AP), and intestinal malrotation.

## Case Report

A term female neonate, weighing 2.63 kg, was delivered in our hospital and was diagnosed with EA/TOF at birth. The antenatal investigations were normal. It appeared as a routine EA/TOF case. She was stabilized, investigated, and underwent primary repair of the EA/TOF on day 3. An extrapleural approach through a right-sided thoracotomy was used. Following ligation of the TOF, the two esophageal ends were mobilized and anastomosed end-to-end with interrupted sutures. Abnormal large arterial vessels were seen coursing across the surgical fields; however, this abnormal anatomy did not interfere with the surgery.

**Fig. 1:**
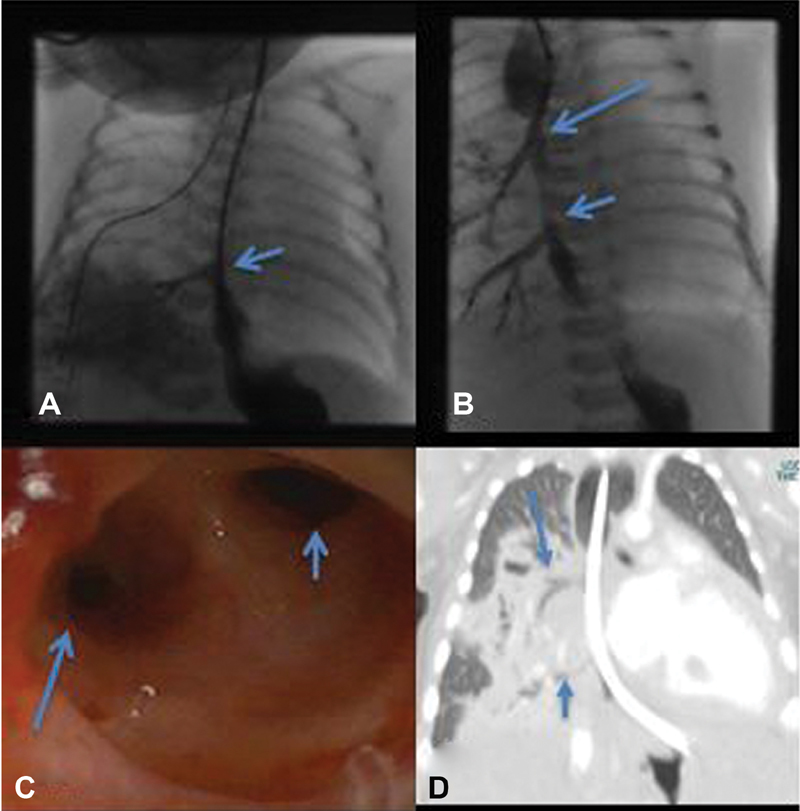
( **A** ) Upper gastrointestinal (GI) contrast series shows reflux of contrast from stomach into the communication (arrow) between the right lung lower lobe and esophagus at T8-T9 level. These images were taken for bilious aspirates on day 8 of life and contrast was instilled via nasogastric tube. ( **B** ) Upper GI contrast series after the second surgery with oral contrast shows a second communication (long arrow) from the esophagus into the right lower lobe at T5-T6 level. Short arrow indicates communication at T8-T9 level. ( **C** ) Esophagoscopy image taken at the level of esophageal anastomosis shows the second communication from the esophagus into the right lower lobe at T5-T6 level (long arrow) and esophageal lumen in the right upper corner (short arrow). ( **D** ) A coronal section of computed tomography scan of chest wall shows the communications from esophagus to right lower lobe at T5-T6 level (long arrow) and T8-T9 level (short arrow).

The patient developed bilious aspirates on day 8. We did an upper gastrointestinal (GI) contrast series that suggested a duodenal obstruction. In the same series, a communication between the right lung lower lobe and esophagus was identified at T8-T9 level ( Fig. 1A ). The patient underwent an exploratory laparotomy on day 9. Intraoperatively, she was found to have DA/AP and intestinal malrotation. She subsequently underwent a Ladd's procedure and a gastrojejunostomy to bypass the duodenal obstruction. A typical duodenoduodenostomy could not be performed as the two atretic ends of the duodenum were far apart. A repeat contrast swallow performed 5 days later confirmed the patency of the esophageal and gastrojejunal anastomosis but also identified a second communication from the esophagus into the right lower lobe at T5-T6 level ( Fig. 1B ). This created a diagnostic dilemma in the form of a recurrent TOF.

The postoperative course in the neonatal unit was also difficult, the infant did not tolerate nasogastric feeds secondary to severe gastroesophageal reflux, and therefore she was given nasojejunal feeds instead. She was initially ventilator dependent and on high flow oxygen in the latter period. The child also underwent esophagoscopy and bronchoscopy during the 5th and 7th week of life. The bronchoscopy findings were normal demonstrating no recurrent TOF, but the esophagoscopy showed an opening, discharging purulent material corresponding to the second communication from the esophagus into the right lower lobe at T5-T6 level ( Fig. 1B and C ) and a patent esophageal anastomosis. Subsequently computed tomography (CT) thorax confirmed two separate communications between the esophagus and right lung tissue, with an accompanying anomalous blood supply ( Fig. 1D ).

**Fig. 2:**
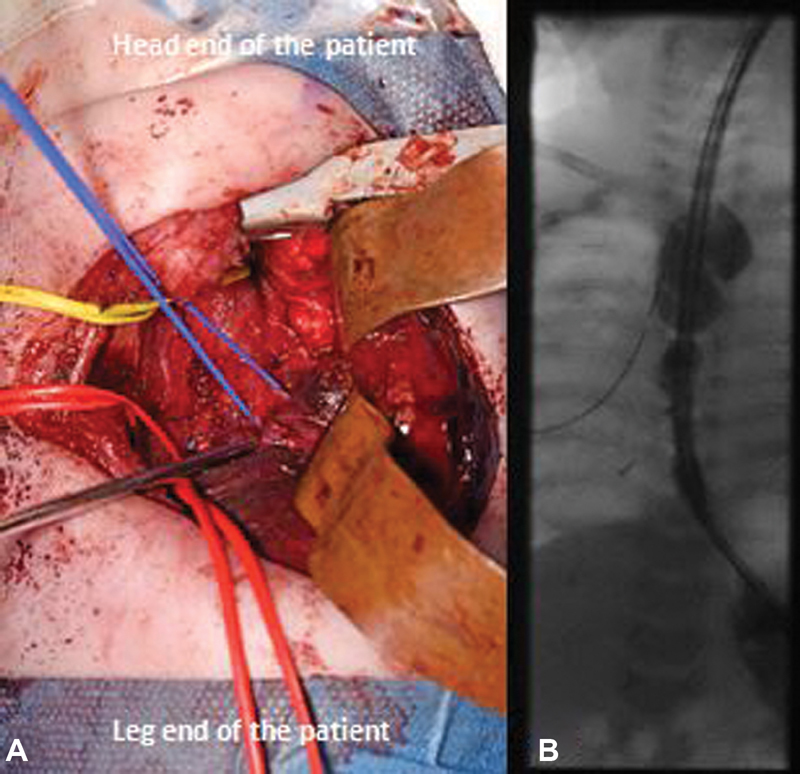
( **A** ) This is the surgeon's view of posterolateral incision of right thoracotomy. Yellow loop encircles the esophagus. The esophageal anastomosis was intact. Red vessel loop encircles the anomalous vessels supplying to right lower lobe. Blue loop encircles esophageal connection that enters into the right lower lobe which is being retracted by retractors. ( **B** ) Postoperative upper gastrointestinal contrast image after definitive surgery.

At exploratory thoracotomy at 4 months of age, a three-lobed right lung was seen; however, the lower lobe was enlarged and connected in two separate locations to the esophagus. The right lower lobe was separated from the esophagus, by ligating and dividing these two fistulous connections, which bore more resemblance to bronchial type tissue, followed by a partial lobectomy ( Fig. 2A ). A postoperative upper GI contrast series identified a patent esophageal anastomosis, no leakage, and no fistulous connections ( Fig. 2B ). The child required postoperative ventilation for 12 days. The chest drain was inserted intraoperatively. It was removed on 20th postoperative day as there was chest infection with suspicion of collection. She was also given a prolonged course of antibiotics according to culture and sensitivity reports of chest drain fluid and stopped after removal of chest drain. The child was discharged on postoperative day 41 with full oral feeds at 5 months of age. She is currently feeding well and gaining weight as per the last follow-up that was done at 6 months postoperatively.

## Discussion

The term bronchopulmonary foregut malformation was first used by Gerle et al in 1968. 4 It was used to denote extralobar or intralobar sequestrations in communication with the foregut. CBPFM are extremely rare anomalies. 1 It is characterized by a congenital and patent communication between the respiratory tract and foregut. They arise due to defective budding and separation of the primitive foregut. Developing lung tissue joins the esophagus through a focal mesodermal defect. This later gets separated from normal developing lung by the rapidly growing esophagus. 5

According to the classification proposed by Srikanth et al, our case belonged to Group IB and is very rare. 3 Yang et al 1 have described a total of 61 cases of CBPFM after reviewing the available literature of which only four cases (6.6%) belonged to group IB. It is more common in females and on the right side, as in our case. 1

Most of these cases were diagnosed in the 1st year of life. The neonatal diagnosis is difficult. 2 The symptoms in group I patients will be due to EA + TOF and present with drooling of saliva, failure to pass a nasogastric tube or choking on feeds. The children in other groups can have persistent cough, repeated aspirations or choking with feeds, recurrent respiratory tract infections, and failure to thrive. 1 Our case had all the above features along with a constant need for respiratory support in terms of invasive and noninvasive ventilation in the initial and latter stages of hospital stay, respectively. Hemoptysis, nocturnal cough, and epigastric pain have also been described as presenting features. 1 2

CBPFMs are also known to be associated with other congenital malformations including EA + TOF, cardiovascular anomalies, VACTERL associations, skeletal malformations, anorectal malformations, and diaphragmatic hernias. 1 2 Our patient had DA/AP and intestinal malrotation that have never been reported before with CBPFM.

The diagnosis of group I CBPFM can be difficult due to its association with EA + TOF. As in our case, most of the group I CBPFMs are initially operated for EA + TOF. 1 2 Persistent atelectasis of one lung or opacity of the hemithorax, refractory respiratory distress, and feeding difficulties will prompt further investigations. Chest X-rays can show a hazy hemithorax, mediastinal shift, hypoaerated or normal lungs. 2 Upper GI contrast series has been shown to be confirmatory in most of cases. A CT scan and bronchoscopy aided the diagnosis in other cases. 1 2 In our case, it was an upper GI contrast series undertaken in light of bilious aspirates after initial surgery for EA + TOF that revealed the CBPFM and DA/AP. The second contrast study done after surgical repair of the duodenal atresia added to the dilemma after it showed another connection between the esophagus and the right lung, raising the suspicion of a recurrent TOF. Bronchoscopy with esophagoscopy and a CT scan helped us to establish the final diagnosis of group IB CBPFM with two communications between the esophagus and the right lung.

Surgery has been described as the only option for treating this condition definitively 1 2 involving thoracotomy and dividing the communications between the esophagus and lung. These anomalies are known to be supplied by large anomalous blood vessels arising from the systemic or pulmonary artery. 1 In our case, there was a large anomalous artery arising directly from the descending thoracic aorta and supplying the CBPFM. We would recommend preoperative CT scan to identify these anomalous vessels to plan adequately for surgery.

To conclude, CBPFM are extremely rare anomalies and present with respiratory and feeding difficulties. Early diagnosis and recognition of associated malformations can influence the outcome. It requires a high index of suspicion with the use of an upper GI contrast series to delineate the abnormal communications with the esophagus. The treatment of choice is resection of the affected lung and disconnection of the esophageal communications.

## Notes

New Insights & the Importance for the Pediatric Surgeon Communicating bronchopulmonary foregut anomalies can be easily missed. The diagnosis once confirmed, we need to optimize the patient for definitive surgery; otherwise, the outcome can be fatal. The index case also had multiple other anomalies and this makes the case unique.
